# Supplementary material for: Finding the balance between model complexity and performance: Using ventral striatal oscillations to classify feeding behavior in rats
Source: PLoS Comput Biol. 2019 Apr 22;15(4):e1006838. doi: 10.1371/journal.pcbi.1006838 (PMC6497302; doi:10.1371/journal.pcbi.1006838)
Supplement: S1 Text — (DOCX) [file pcbi.1006838.s001.docx]

**SUPPLEMENTAL METHODS**

**Behavioral measures**

Since not all recordings were exactly 2 hours, the behavioral timestamps were normalized by the session length, allowing for direct comparison across sessions. Once timestamps were normalized, the percent of animals engaged in a given behavior at a given point in time could be calculated. For visualization, these percentages were divided into ten segments corresponding to 10% of the length of a session.

**Signal processing**

The only difference in signal processing from our previous work ([[1](#_ENREF_1)]) was using a 256 sample (~0.6 second) Hamming window to calculate power. Although whole session analysis was normalized differently, within session analysis used the same normalization as Doucette et al. ([[1](#_ENREF_1)]).

**Chewing artifacts**

To minimize the chances of chewing artifact contaminating the models, two features that were possibly influenced by chewing were removed from all model building. To determine if a feature was possibly influenced by chewing the percent change in each feature from Base to Dep24 and Dep48 was regressed against the percent change in voracity from the same recordings. Voracity was calculated as ratio between grams of food consumed to time spent eating in seconds (Figure S1A). Out of the 60 regressions, two had uncorrected p-values <.05 and were removed from analysis (Figure S1B).

**Effect size**

The Mann-Whitney *U* test statistic was converted to an r-family effects size (Eq. 1; [[2](#_ENREF_2)]), and then Cohen’s *d* (Eq. 2; [[3](#_ENREF_3)]).

$r=\frac{z}{\sqrt[2]{N}}$ (Equation 1)

$d=\frac{2r}{\sqrt[2]{1-r^{2}}}$ (Equation 2)

**Determining the optimal number of bins for model training**

To train a behavior classifier model each 5-second bin was represented as a single dependent variable (that bin’s behavioral category) and 58 predictor variables (normalized LFP features). Each session provided over 1000 bins allowing the creation of independent training- and test-sets by segregating ~20% of the bins into the naïve test-set such that the original ratio of bins per behavior for that session was maintained (i.e., if a given session had 100 feeding and 900 not-feeding bins, then the naïve test-set would have a random sample of 20 feeding and 180 not-feeding bins). As feeding bins were relatively rare (on average 18% of bins) and rare event detection can lead to over-fit models or majority classifiers, two methods for dealing with rare events were tested: imputation and weighting. For imputation the adaptive synthetic sampling approach for imbalanced learning (ADASYN; [[4](#_ENREF_4)]) was used and for weighting the ratio of the two behaviors was used to compute weights (e.g., if there were twice as many not-feeding bins, then the feeding bins were given twice the weight). For imputed and weighted models the number of bins in the training set was slowly increased to create learning curves with the imputed models achieving stability with smaller training sets than the weighted models (Fig S2A-B), so, when needed, ADASYN was implemented. To determine the number of bins (sample size) at which model performance became stable, each sample size was compared to the performance of the model trained from the largest sample size using two-sample two-tailed t-tests and p-values were adjusted for multiple comparisons using the Bonferroni correction. By training models with samples sizes indicative of stable performance then allowed comparison between models using different sample sizes without worrying that any differences were merely due to more or fewer samples.

**Pre-feeding vs. not-feeding**

Pre-feeding vs. not-feeding models were trained using the lasso algorithm with 250 total samples (imputed with ADASYN to well above the minimum suggested by the learning curves; Fig S2). Imputation was done on ~80% of the data, after ~20% of the data were set aside for the test-set, to prevent overfitting. The training data were balanced with 125 pre-feeding bins and 125 feeding bins while the test-sets matched the ratio of pre-feeding to feeding bins seen in the recordings.

**Feeding vs. not-feeding**

ADASYN was used to impute the feeding bins data up to 250 bins per animal for training after removing ~20% of the data for testing. A randomly selected subset of 250 not-feeding bins per animal was also used for the training set (500 bins total per animal).

**Complexity vs. performance**

*Individuals*

To test the performance of models built from an individual animal, training sets of 500 bins were used ensuring that the models had attained a stable performance and thus comparable to models built with data from more animals. As before, imputation with ADASYN occurred after leaving aside ~20% of each animal’s data. Further, the same 20% of each animal’s data was used for the test-sets such that the data used to test an individual model would be a part of the test-set used for the population models.

*Conditions*

When assessing the importance of including data from multiple conditions in the training data, 4000 bins from each condition (500 bins from the 8 animals that had data from all conditions) were used. These sample sizes allowed for the evaluation of population-based models or individualized models.

**SUPPLEMENTAL REFERENCES**

1. Doucette WT, Dwiel L, Boyce JE, Simon AS, Khokhar JY, et al. (2018) Machine learning based classification of deep brain stimulation outcomes in a rat model of binge eating using ventral striatal oscillations Front Psychiatry.

2. Fritz CO, Morris PE, Richler JJ (2012) Effect size estimates: current use, calculations, and interpretation. J Exp Psychol Gen 141: 2-18.

3. Friedman H (1968) Magnitude of experimental effect and a table for its rapid estimation. Psychological Bulletin 70: 245-251.

4. He H, Bai Y, Garcia EA, Li S (2008) ADASYN: Adaptive Synthetic Sampling Approach for Imbalanced Learning. Proceedings of the International Joint Conference on Neural Networks. pp. 1322-1328.
