# Supplementary figures and images for: Finding the balance between model complexity and performance: Using ventral striatal oscillations to classify feeding behavior in rats
Source: PLoS Comput Biol. 2019 Apr 22;15(4):e1006838. doi: 10.1371/journal.pcbi.1006838 (PMC6497302; doi:10.1371/journal.pcbi.1006838)

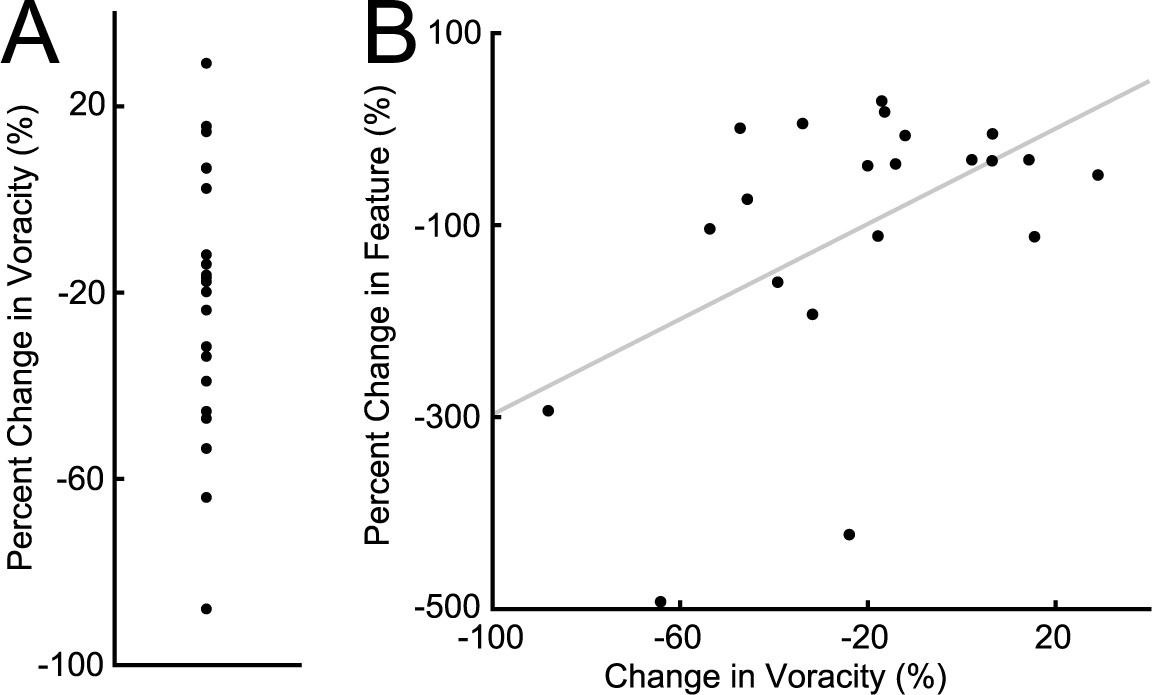

Supplement: S1 Fig — Features potentially contaminated by chewing noise A. Distribution of percent changes in voracity from baseline to food deprived conditions (Dep24 and Dep48). B. Regression between percent change in shell left core left theta coherence and percent change in voracity from baseline to food deprived conditions; p < 0.01; R2 = 0.3. (TIF) [file pcbi.1006838.s002.tif]

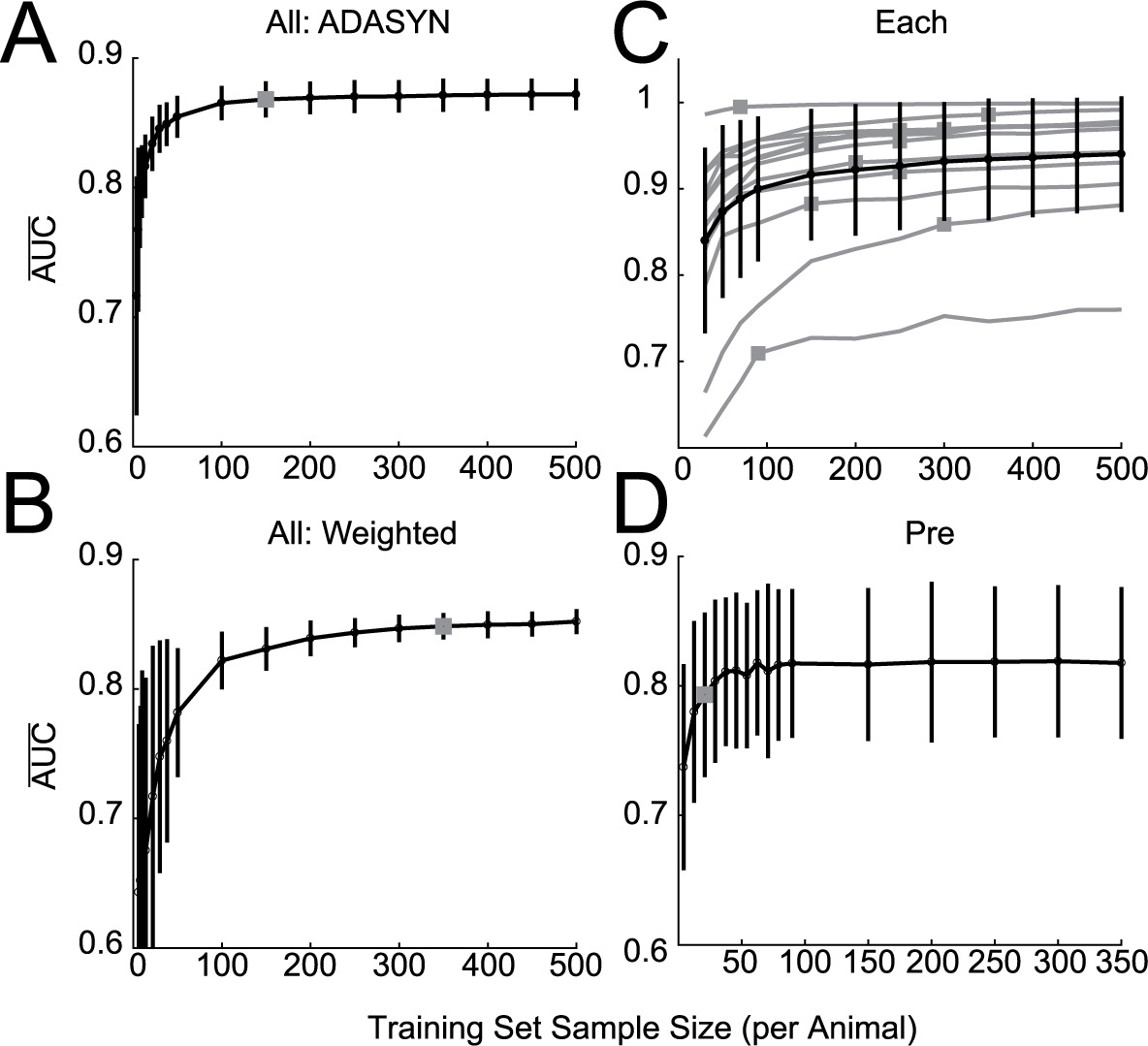

Supplement: S2 Fig — Circles indicate average AUC and vertical bars represent 95% confidence intervals. Grey boxes indicate at which trial number performance becomes insignificantly different from performance of models with most data. A. Testing population models using ADSYN imputation and between 6 and 500 trials per animal; 150 per animal (1800 total) are needed to achieve performance of models using all 500 per animal (6000 total). B. Testing population models using weighted outcome variables and between 6 and 500 trials per animal; 350 trials per animal (4200 total) would be needed to match performance of full models. C. Testing individual models (grey lines) using between 30 and 500 trials; the most any individual needed was 350 trials to match the performance of models made with all 500 trials. D. Testing pre-feeding models using between 4 and 350 trials each; 13 trials per animal were needed to match the performance of 350 trials per animal. (TIF) [file pcbi.1006838.s003.tif]

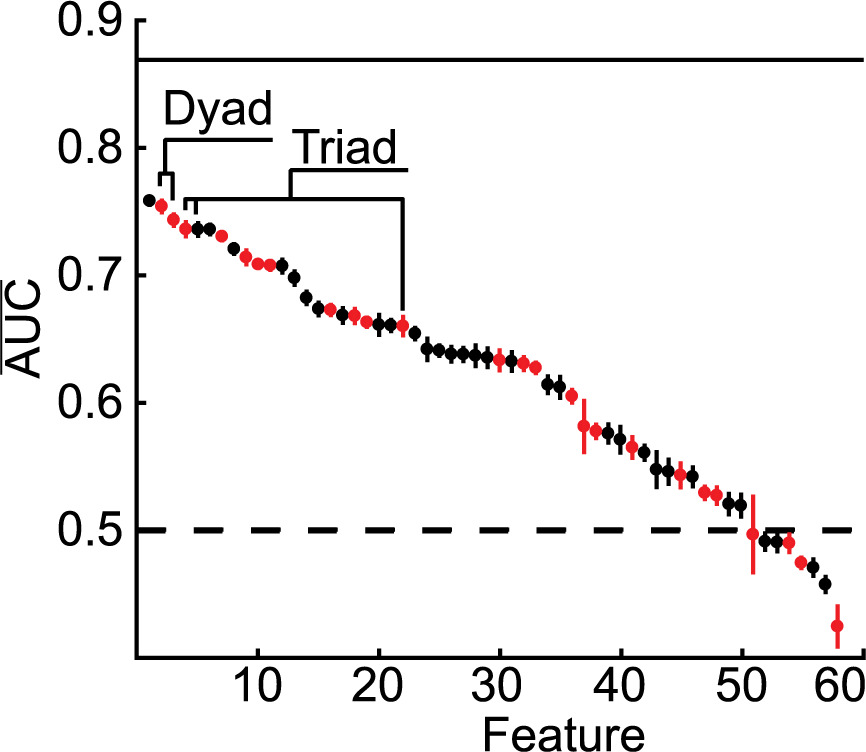

Supplement: S3 Fig — Average AUC indicated by a circle and 95% confidence interval by a vertical bar. (TIF) [file pcbi.1006838.s004.tif]

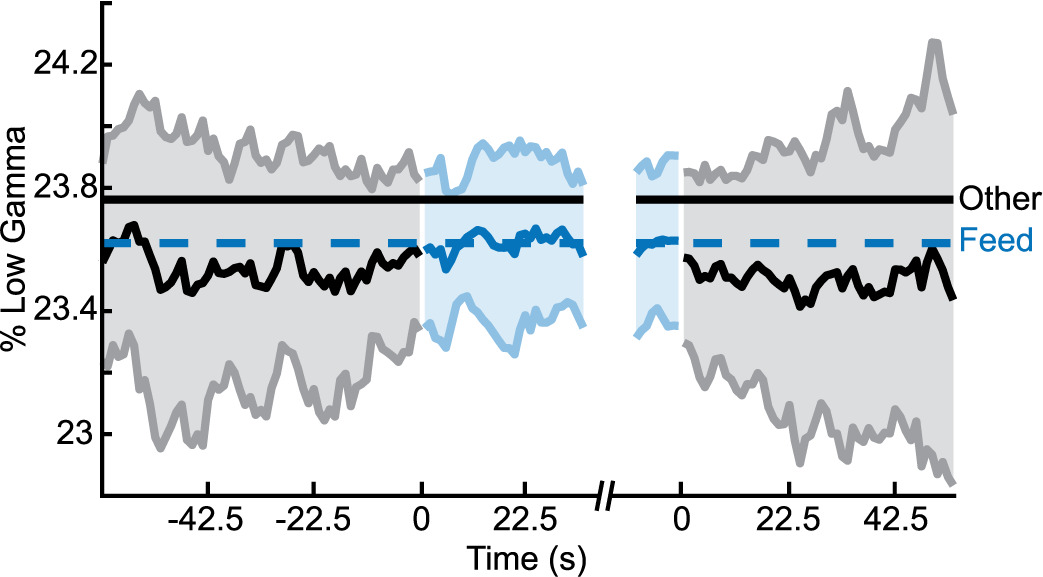

Supplement: S4 Fig — Power was normalized by total power of shell right and averaged across all trials and animals around the beginning and end of feeding epochs. Shading indicates ±1 standard deviation. Black traces represent averages before and after feeding and blue traces represent averages during feeding. Dashed blue and solid black lines indicate average feature activity for either feeding trials or all other trials outside of these plots. (TIF) [file pcbi.1006838.s005.tif]
