# Supplementary material for: Finding the balance between model complexity and performance: Using ventral striatal oscillations to classify feeding behavior in rats
Source: PLoS Comput Biol. 2019 Apr 22;15(4):e1006838. doi: 10.1371/journal.pcbi.1006838 (PMC6497302; doi:10.1371/journal.pcbi.1006838)
Supplement: S1 Table — Locations: Sell left (SL), shell right (SR), core left (CL), core right (CR). Frequencies: delta (d), theta (t), alpha (a), beta (b), low gamma (lg), and high gamma (hg). Power features have one location and frequency (e.g. shell right alpha power is SRa) and coherence have two locations and one frequency (e.g. high gamma coherence between shell left and core right is SLCRhg). Feature order (Feature 1 vs. Feature 2) is not important. (DOCX) [file pcbi.1006838.s006.docx]

| Monad | | Dyad | | | Triad | | | |
| --- | --- | --- | --- | --- | --- | --- | --- | --- |
| Feature 1 | AUC | Feature 1 | Feature 2 | AUC | Feature 1 | Feature 2 | Feature 3 | AUC |
| SRa | 0.685 | SRa | SRhg | 0.724 | SLhg | SRa | CLd | 0.748 |
| SRhg | 0.683 | SRhg | CRa | 0.720 | SRhg | CRa | CRhg | 0.747 |
|  |  | SRa | CLd | 0.719 | SRd | SRa | SRhg | 0.746 |
|  |  | SLb | SRhg | 0.719 | SRhg | CLa | CLhg | 0.745 |
|  |  | SRd | SRa | 0.716 | SRa | SLSRlg | SRCRlg | 0.744 |
|  |  | SRa | CRd | 0.716 | SLa | SLSRlg | SLCRlg | 0.743 |
|  |  | SRa | SLCRhg | 0.715 | SRa | CRd | SLSRlg | 0.742 |
|  |  | SLa | SRhg | 0.715 | SRa | SLSRlg | SLCRlg | 0.742 |
|  |  |  |  |  | SRa | SRhg | CLd | 0.742 |
|  |  |  |  |  | SLb | SRhg | CLd | 0.742 |
|  |  |  |  |  | SLhg | CLhg | CRa | 0.741 |
|  |  |  |  |  | SLb | SLSRlg | SLCRlg | 0.741 |
|  |  |  |  |  | SRa | SLSRlg | SLCRhg | 0.741 |
|  |  |  |  |  | SLhg | SRd | SRa | 0.74 |
|  |  |  |  |  | SLb | SRhg | CLhg | 0.74 |
|  |  |  |  |  | CLd | CLa | SLSRlg | 0.74 |
|  |  |  |  |  | SRa | SRhg | CRd | 0.740 |
|  |  |  |  |  | SLa | SRhg | CLd | 0.740 |
|  |  |  |  |  | SLb | SLhg | CLd | 0.739 |
|  |  |  |  |  | SRa | SRhg | CLhg | 0.739 |
|  |  |  |  |  | SLhg | SRa | CRd | 0.739 |
|  |  |  |  |  | SRa | CLd | SLSRlg | 0.739 |
|  |  |  |  |  | SRa | SRhg | SLCRhg | 0.738 |
|  |  |  |  |  | SLhg | SRa | SLCRlg | 0.738 |
|  |  |  |  |  | SLa | SLhg | CLd | 0.738 |
|  |  |  |  |  | SLa | SRhg | CRd | 0.738 |
|  |  |  |  |  | SLhg | SRa | SRCRlg | 0.738 |
|  |  |  |  |  | SLhg | CLhg | SLCRlg | 0.738 |
|  |  |  |  |  | SLa | SRhg | CLhg | 0.738 |
|  |  |  |  |  | SLa | SLSRlg | SRCRlg | 0.738 |
|  |  |  |  |  | CLa | SLSRlg | SLCRlg | 0.737 |
|  |  |  |  |  | SRa | SLSRlg | SRCRhg | 0.737 |
|  |  |  |  |  |  |  |  |  |
